# Pre and post-operative serum levels of titanium cobalt and aluminium after implant placement

**DOI:** 10.6026/973206300200690

**Published:** 2024-06-30

**Authors:** Richa Jain, Bhumika Sehdev, Pratik Chaudhari, Sanjukta Panda, Binoo Verma, Supriya Mishra, Mahesh Ghadage, Miral Mehta

**Affiliations:** 1 Department of Periodontology, College of Dental Sciences and Hospital, Rau, Indore, M.P., India; 2 Department of Periodontology, RKDF Dental College and Research Center, Bhopal, M.P., India; 3 Kalinga Institute of Dental Sciences, KIIT Deemed to be University, Bhubaneswar, Odisha, India; 4 Department of Prosthodontics Crown & Bridge and Implantology, Bhabha College of Dental Sciences, Bhopal, M.P., India; 5 Government Dental College, Raipur, Chhattisgarh, India; 6 Department of Prosthodontics and Crown & Bridge, Bharati Vidyapeeth (Deemed to be University) Dental College and Hospital, Navi Mumbai, India; 7 Department of Pedodontics, Karnavati School of Dentistry, Karnavati University, Gandhinagar, Gujarat, India

**Keywords:** Dental implants, serum, titanium, cobalt, aluminium

## Abstract

The preoperative serum levels and postoperative serum levels of titanium, cobalt and aluminium from dental implants in order to assess the release of these ions and to assess any risk of toxicity from these ions after dental implant placement is of interest to dentists. It was observed that there was very slight increase in serum concentration of titanium, cobalt and aluminium after 12 months of placement of implants as compared to before placement of implants. However the increase was non-significant statistically. Our study concluded that the use of dental implants does not pose any risk of toxicity of metal ions like titanium, aluminium and cobalt because of very slight non-significant increase in serum levels of these ions 12 months after implant placement.

## Background:

In the past 20 years, the application of dental implants to replace missing teeth has grown in significance, and these days, implants are the main treatment for edentulism [1, 2-3]. Since titanium has excellent mechanical qualities, a relatively lower density, and the capacity to osseointegrated, it is the material preference for the majority of implants utilized nowadays [4, 5, -6]. Nevertheless, concerns about metal ion leakage from implants and their possible medical consequences have always been expressed [7, 8- 9]. Numerous writers have investigated how different orthopedic and dental implants produce titanium. Individuals who underwent total hip arthroplasty showed considerably higher concentrations of titanium, chromium and cobalt than controls, according to a ten-year follow-up study [10, 11- 12]. Despite being categorized as a bio inert substance, research has shown that blood can however include titanium from orthopedic implants [7, 8- 9]. Nevertheless, there has been little discussion of the precise metal ion emission from dental implants in humans as well as animal research [12, 13- 14]. Pure titanium (cpTi) as well as Ti-6Al-4V is the two titanium alloys that are currently marketed; over a ten-year period, both alloys have demonstrated excellent clinical rate of success of as high as 99 percent [11, 12, 13, 14, 15, 16- 17]. In addition to aiding in osseointegration, which is necessary for the long-term durability of the implants, both alloys remain biocompatible with native tissues once they come into touch with bone and gingival tissues [9-14]. Implant-related releases of metallic ions could be caused by a number of processes. These consist of electrochemical deterioration, structural wear, and pitting corrosion, which is a hybrid of the two [10-17]. The amount of metal particles and ions discharged from implants as well as restorations can also be influenced by mechanical elements like fluorides and the micro-gap. Saliva, germs, and substances that have the ability to degrade the titanium oxide layer can come into contact with implant surfaces and restorations, starting corrosion cycles [15, 16, 17-18]. Hydrogen peroxide and fluorides, two therapeutic compounds, can accelerate the deterioration of titanium dental implants and abutments, causing the discharge of harmful ions [19, 20-21]. Conversely, another study found no statistically significant rise in serum or urine titanium levels prior to or following implant insertion in rabbits [17-22]. These days, the majority of dental implants use alloys made of aluminum and cobalt to enhance their mechanical qualities [15- 18]. Therefore, in our study we compared the preoperative serum levels and postoperative serum levels of titanium, cobalt and aluminium from dental implants in order to assess the release of these ions and to assess any risk of toxicity from these ions after dental implant placement.

## Methodology:

## Study participants:

Patients who visited our facility and to replace the lost teeth served as the study's subjects.

## Qualifications for inclusion:

1. Individuals needing to have a single lost tooth in the upper central incisor replaced

2. Individuals in the 18-71 year age range

3. Individuals in good health who do not have any underlying illnesses

4. Individuals with sufficient bone volume to accept a suitably sized implant

5. Patients who are cooperative and ready for the procedure and appropriate aftercare.

## Criteria for exclusion:

1. Individuals with underlying health issues

2. Individuals with harmful behaviors like chewing tobacco or smoking.

3. Individuals with paranormal routines

4. Individuals who needed more than one implant, or who had already had orthopedic or dental implants placed.

To evaluate the condition of the bones, pre-treatment CT (computed tomography) scans were performed on each patient. Based on Misch's categorization for density of bone [5], the implant receiving bone region was categorized, and only instances with Grade D2 were included in the study to guarantee consistency in the condition of the bone.

## Sample size:

There were 60 participants in our study, 36 of whom were men and 24 of whom were women.

## Specimen gathering and examination:

Serum samples were taken from every individual before the placement of implants in order to determine baseline levels of titanium, cobalt, and aluminum ions. Each patient had 10 milliliters of blood taken into sterile containers without the addition of an anticoagulant. Serum was extracted from the blood by centrifugation it after it had close strictly for 20 minutes at room temperature. If the blood had been left to clot longer, there was a chance of external contamination. With a Perkin Elmer optimum 5300 DV ICP-OES apparatus (ICP-OES, ELAN DRC II, Perkin Elmer, SCIEX, Inc.), the samples were examined for titanium, cobalt, and aluminum using Inductively Coupled Plasma Optical Emission Spectrometry (ICP-OES). After that, flapless implant placement was performed. A Micro-Textured surface (MTX) tapered screw-vent implant with hydroxyapatite coating around the middle was used in all patients. Zinzer Inc. Six months later, the implant was loaded.

## Follow up:

After implant implantation, all patients were contacted back six weeks, three months, six months, and twelve months later. Every visit involved the collection of blood samples, serum extraction, and above-mentioned metal ion analysis.

## Statistical analysis:

The Kolmogorov-Smirnov test was utilized to determine the normality of the gathered data. For each patient group, the mean, standard deviations and median were determined. The paired t-test for small samples was used for the statistical assessment of parameters because the datasets were interconnected. The Mann-Whitney U test would have been used if the data weren't normal. The SPSS program, version 21, was used for all statistical analyses (IBM Corp).

## Results:

There was very slight increase in serum concentration of titanium after 12 months of placement of implants (2.40±0.69) as compared to before placement of implants (2.39 ±0.70)(Table 1).

The serum concentration of aluminium before implant placement was 4.16±0.91mg/dl while it was 4.18±0.92 mg/dl 12 months after placement of dental implants there was slight increase in serum levels of aluminum 12 months after implant placement (Table 2).

When compared to preoperative serum levels of cobalt before implant placement (1.28±0.10), the serum levels of cobalt postoperatively 12 months after implant placement was (1.30±0.49) showing marginal increase (Table 3).

## Discussion:

This study was carried out to compare the preoperative serum levels and postoperative serum levels of titanium, cobalt and aluminium from dental implants ions in order to assess the release of these ions. It was observed that there was very slight increase in serum concentration of titanium, cobalt and aluminium after 12 months of placement of implants as compared to before placement of implants. However the increase was non-significant statistically. According to another study which compared metal ion levels in different surgical methods found that a single participant in the dental implant category experienced elevated serum levels of titanium, and another individual had elevated serum levels of both titanium and chromium [12-18]. So of right now, very few studies has measured the concentrations of all three metal ions-chromium, titanium, and aluminum-in the blood of individuals who had implants placed [11-19]. Ion levels in blood were measured in this investigation using absorption spectrometry (AS), and no discernible change in these levels was seen between patient pre- and post-procedure. ICP-OES was the approach employed in this investigation, and it is thought to be better than AS [13-16].

In spite of this our study did not discover a discernible variation in metal ion concentrations before to and following implant implantation. The levels of metal ions increased, but not significantly. The other potential sources of elevated serum levels of aluminum and titanium were not considered in this investigation [14-17]. Large concentrations of titanium are known to be present in processed foods; trace levels can also be detected in soil, drinking water, and the air. Aluminum is more common and can be absorbed through the air, water and soil [15- 19]. Exposure to aluminum is further increased by the preservatives added to processed foods and the container that houses them [14-16]. Each participant in this study acted as their own control to prevent confounding. Two factors make implant/superstructure systems' galvanic corrosion significant: (1) the potential for biological impacts associated with the disintegration of alloy elements; and (2) the potential for bone deterioration resulting from current flow resulting from galvanic corrosion [15- 19]. Ti alloys' ability to withstand corrosion is dependent on an oxide coating (TiO2) known as the "passive layer," which can be disrupted to liberate ions [22, 23, 24-25]. The most often utilized type of titanium is titanium dioxide. Percutaneous as well as permucosal exposure to titanium is increased by the quick growth of goods containing titanium. The allergy to titanium is lower than that to other metals [20, 21, 22-23]. Prior to implant implantation, it is recommended to inquire about signs of hypersensitivity from the patients, and patch evaluations can also be done on those who have previously experienced hypersensitivity reactions [24,25]. It has been demonstrated that lower titanium escapes from titanium alloys than from commercially purest titanium, and that the quantity of corrosion can be determined by the material utilized for implants [14-18]. The overall implant-bone surface area did not influence the quantity of implants, and gender of the individual had no bearing on the serum Ti levels [19-23]. Research has indicated that the implant's diameter and overall area had minimal bearing on the amount of Ti released into the bone [21, 22, 23, 24, 25-26]. The dimensions of the particles affect what happens to the metal ions that are produced in this way. Particles with a smaller size can either be swallowed by macrophages or spread through lymphatics to organs like bone marrow, liver and spleen, while bigger particles might stay around the implant [17-19]. Metallic ions discharged from the implants are likely to enter the bloodstream and accumulate in the erythrocytes if the corrosion worsens. As a result, monitoring these ion concentrations in blood would provide a reliable indicator of both mechanical as well as chemical implant deterioration [11-18]. Since the surface area of dental implants along with total implant bone is tiny, there is no link between the two. The corrosion products are transported by the circulatory system to the hair, lungs, spleen where they raise serum levels [16-20]. Implant-related metallic ion emission may have localized as well as systemic consequences. Ti ions are frequently detected at the peri-implant tissue level, and they are found to be comparatively higher in peri-implantitis areas than in healthy implants [21, 22]. The material that is most commonly employed in dentistry is titanium, which is connected to antenna activity and may have negative consequences from electromagnetic radiation [21-24]. The degree of titanium corrosion and its correlation with peri-implantitis raise consciousness regarding the connection between periodontitis and peri-implantitis [22-24]. Long-term deterioration may trigger ions to be released into the tissues surrounding the implant surface, disintegrate the implant, and wear down the material, which can result in an implant failure including abutment breakage [23-25]. Titanium has not been found to have any negative effects on human beings, although research on rats has linked titanium to adverse reactions in the lungs [12-17]. However, it has been established that aluminum is hazardous to people. Diabetes encephalopathy, Parkinson's disease, and Alzheimer's disease have all been related to elevated blood levels of aluminum [13-19]. It has been demonstrated that prolonged exposure to aluminum raises the likelihood of fractures with pathology and osteomalacia [20-26]. Elevations in cobalt have been associated with gastrointestinal distress and renal impairment [14-18]. Prior to the study, no effort had been taken to standardize ion concentrations within this group's cross-section. If this standardization were to be used to subsequent research, it would allow for the randomization and employment of a distinct control group, which would reduce bias [16-23]. Tantalum and zirconium are two alternative implant materials that have been available recently in order to avoid titanium toxicity. Nevertheless, titanium has a good long-term success rate when compared to other materials, and its toxicity is limited to the tissues surrounding the implants [17-26]. Patients in this study had a 12-month follow-up period. On the other hand, dental implants are meant to last a lifetime, with an average lifespan of about 40 years. Thus, it's likely that the metallic ion quantities would rise to proportions that could potentially be clinically significant if these patients were monitored for longer periods of time [21-26].

## Conclusion:

Our study concluded that the use of dental implants does not pose any risk of toxicity of metal ions like titanium, aluminium and cobalt because of very slight non-significant increase in serum levels of these ions 12 months after implant placement.

## Figures and Tables

**Table 1:** Preoperative serum levels before implant placement and post -operative serum levels of titanium after implant placement

	**Mean**	**Std deviation**	**Range**	**SEM**	**P value**
Before placement	2.39	0.7	1.22-3.88	0.2371	
After placement					
6 weeks	2.38	0.81	1.24-3.88	0.1389	0.1669
3 months	2.39	0.69	1.13-3.78	0.126	0.1665
6 months	2.39	0.68	1.13-3.78	0.1242	0.1662
12 months	2.4	0.69	1.14-3.78	0.126	0.1221

**Table 2:** Preoperative serum levels before implant placement and post-operative serum levels of aluminium after implant placement

	**Mean**	**Std deviation**	**Range**	**SEM**	**P value**
Before placement	4.16	0.91	2.50-5.65	0.2572	
After placement					
6 weeks	4.16	0.91	2.50-5.64	0.2572	1.1111
3 months	4.17	0.91	2.50-5.61	0.1572	0.1727
6 months	4.19	0.92	2.51-5.65	0.158	0.9968
12 months	4.18	0.92	2.51-5.65	0.158	0.1348

**Table 3:** Preoperative serum levels before implant placement and post-operative serum levels of cobalt after implant placement

	**Mean**	**Std deviation**	**Range**	**SEM**	**P value**
Before placement	1.28	0.1	0.33-2.99	0.3482	
After placement					
6 weeks	1.28	0.61	0.24-2.88	0.1178	0.188
3 months	1.29	0.49	0.13-2.78	0.1159	0.1776
6 months	1.29	0.48	0.13-2.78	0.1121	0.1773
12 months	1.3	0.49	0.14-2.78	0.1472	0.1332
